# Supplementary material for: Identification of disease modules using higher-order network structure
Source: Bioinform Adv. 2023 Oct 4;3(1):vbad140. doi: 10.1093/bioadv/vbad140 (PMC10582521; doi:10.1093/bioadv/vbad140)
Supplement: vbad140_Supplementary_Data [file vbad140_supplementary_data.pdf]

Identification of Disease Modules Using Higher-Order Network Structure
Supplementary Information

Pramesh Singh, Hannah Kuder, Anna Ritz
Reed College, Portland, Oregon, USA

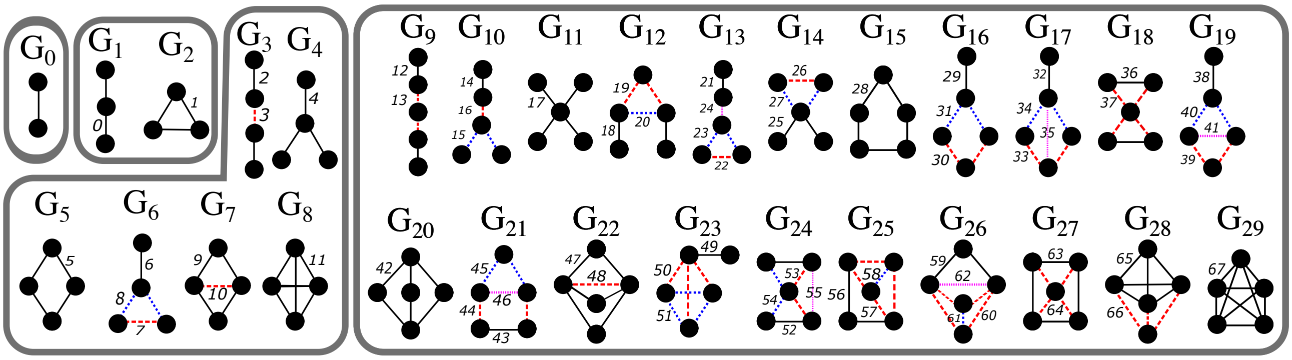

Figure S1: All 30 graphlets up to five nodes with 68 $(0-67)$ edge orbits indicated by different style and colors. Graphlet G_0 is a simple edge and does not have an edge-orbit label. For a given size, the graphlets are ordered by non-decreasing number of edges (form sparse to dense).

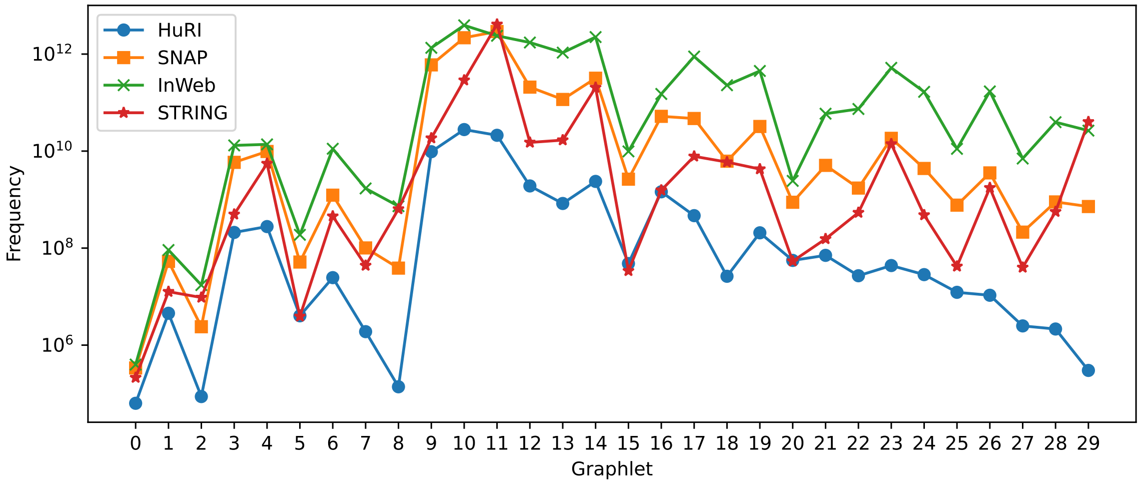

Figure S2: Frequency distribution of different graphlets in all four interactomes considered.

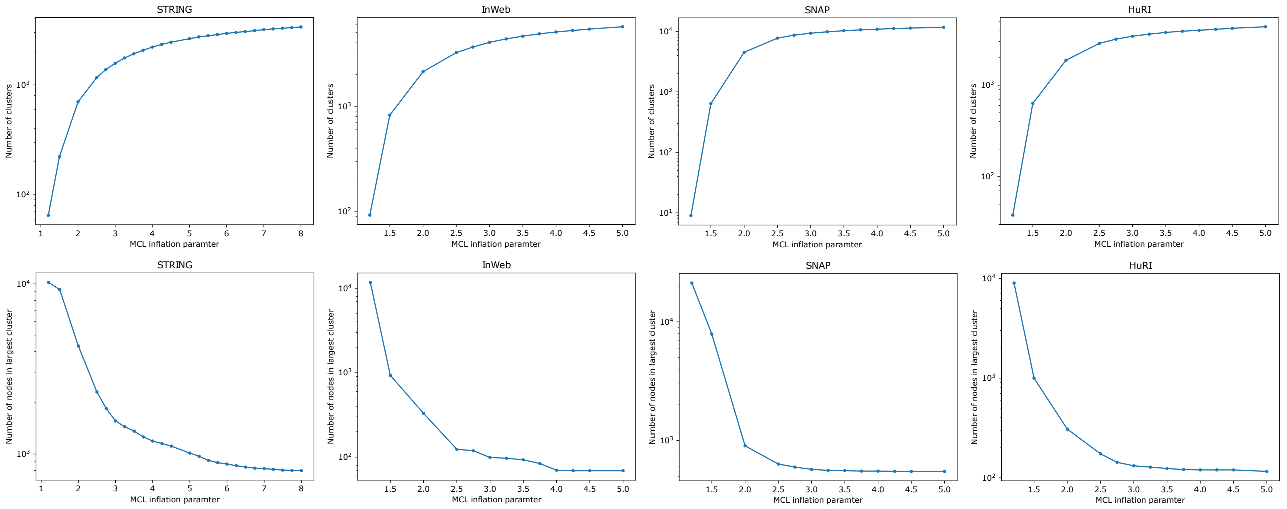

Figure S3: Parameter sweep plots for STRING, InWeb, SNAP, and HuRI. MCL was performed on each interactome's G_0 network for varying inflation values. The first plot for each interactome shows the number of clusters plotted against the inflation parameter used. The second plot for each interactome shows the number of nodes in the largest cluster plotted against the inflation parameter used.

	STRING	InWeb	SNAP	HuRI	SNAP (Windels et al.)
Time to compute edge orbits/	1375.895	10868.054	1255.261	21.584	
Time to compute graphlet adjacency					98115
Time to find G0 clusters	2.954	7.638	23.471	2.562	-
Time to find G1 clusters	2.952	7.651	22.827	2.617	216
Time to find G2 clusters	2.335	6.103	14.821	1.079	-
Time to find G3 clusters	2.944	7.652	22.948	2.543	3912
Time to find G4 clusters	2.961	7.672	22.893	2.589	231
Time to find G5 clusters	1.939	6.768	19.581	2.095	54
Time to find G6 clusters	2.946	7.616	22.834	2.509	214
Time to find G7 clusters	2.309	6.106	14.812	1.075	49
Time to find G8 clusters	1.860	5.084	9.071	0.360	-
Time to find G9 clusters	2.945	7.654	23.736	2.608	
Time to find G10 clusters	2.952	7.656	23.825	2.576	
Time to find G11 clusters	2.978	7.702	24.627	2.550	
Time to find G12 clusters	2.959	7.616	24.061	2.495	
Time to find G13 clusters	2.966	7.636	24.205	2.567	
Time to find G14 clusters	3.033	7.609	23.753	2.492	
Time to find G15 clusters	1.977	6.852	20.842	2.195	
Time to find G16 clusters	2.949	7.752	23.943	2.598	
Time to find G17 clusters	2.974	7.602	23.943	2.452	
Time to find G18 clusters	2.336	6.079	15.747	1.060	
Time to find G19 clusters	2.915	7.607	23.989	2.492	
Time to find G20 clusters	1.786	6.512	20.023	2.078	
Time to find G21 clusters	2.455	7.122	21.039	2.156	
Time to find G22 clusters	2.239	6.082	15.329	1.020	
Time to find G23 clusters	2.921	7.552	22.808	2.325	
Time to find G24 clusters	2.284	6.064	15.624	1.049	
Time to find G25 clusters	2.389	6.986	20.337	1.888	
Time to find G26 clusters	1.835	5.075	9.499	0.362	
Time to find G27 clusters	1.577	5.213	13.161	0.779	
Time to find G28 clusters	1.832	5.074	9.298	0.347	
Time to find G29 clusters	1.548	4.427	5.812	0.122	

Table S1: Runtime in seconds for clustering with respect to different graphlets in four PPIs. Last column shows approximate runtime on SNAP using the method of Windels et al. for up to 4-node graphlets. We did not run this method to obtain G0, G2, and G8-based clusters since the induced networks for these graphlets are identical to our method. All computations were performed on a MacBook Pro with Apple M2 Pro 10-core CPU and 32 GB unified memory.

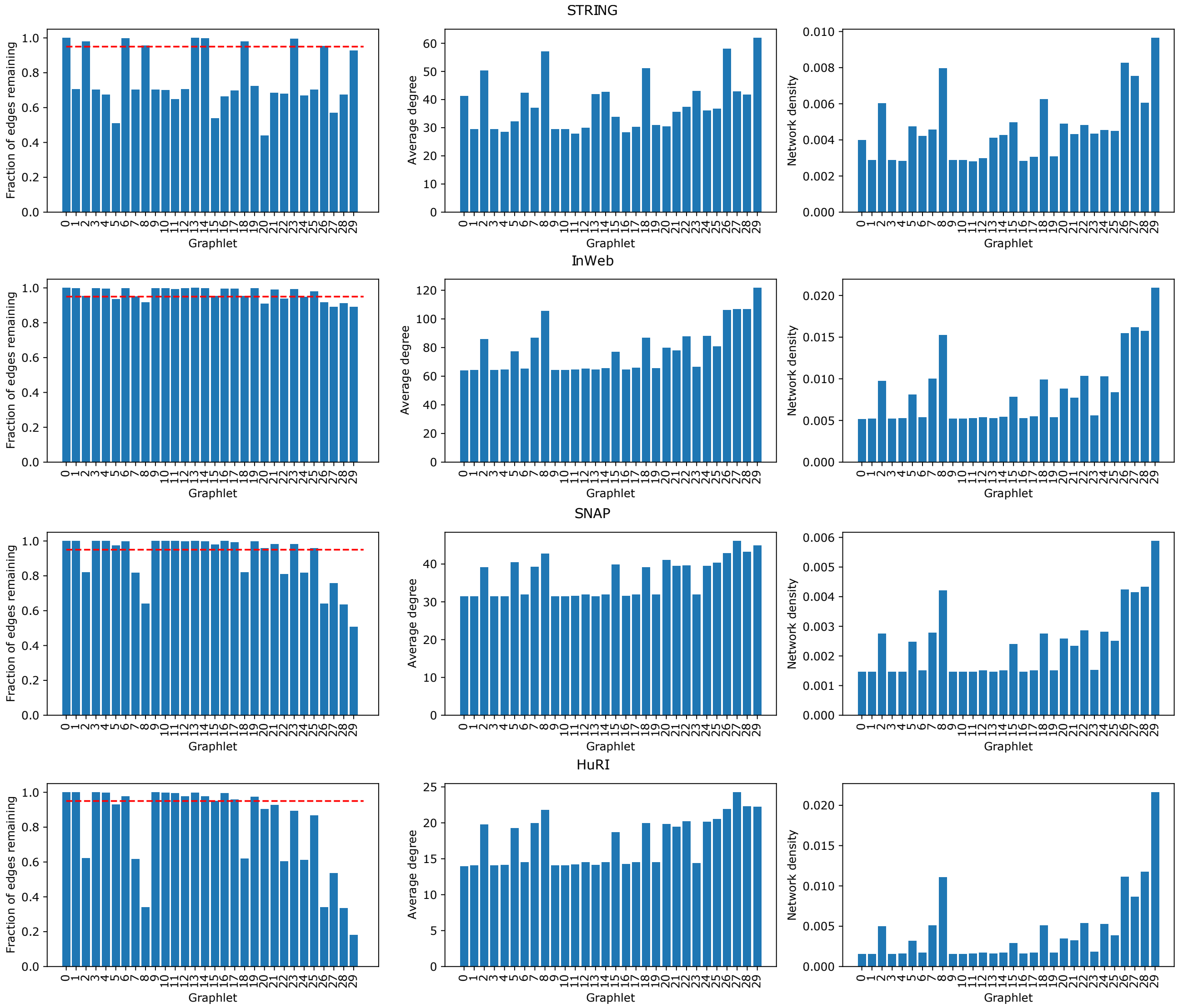

Figure S4: Global properties of modified networks corresponding to graphlets $G_0 - G_{29}$ for all four interactomes considered. The red dashed line represents fraction of edges $= 0.95$ of the original (G_0) network. Networks with the remaining fraction of edges larger than this threshold are considered redundant.

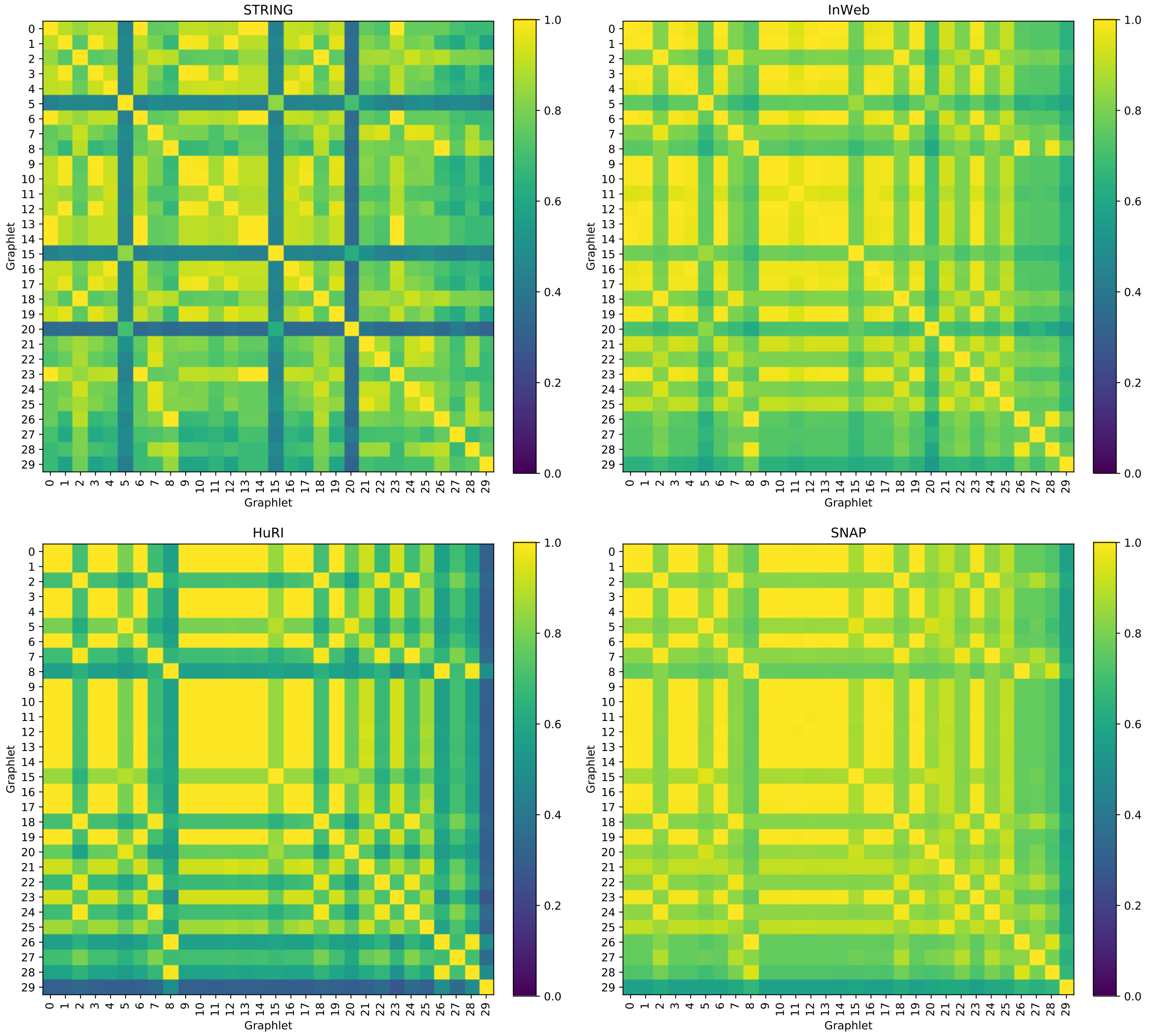

Figure S5: ARI between different graphlets-based clusterings (with respect to all graphlets $G_0 - G_{29}$).

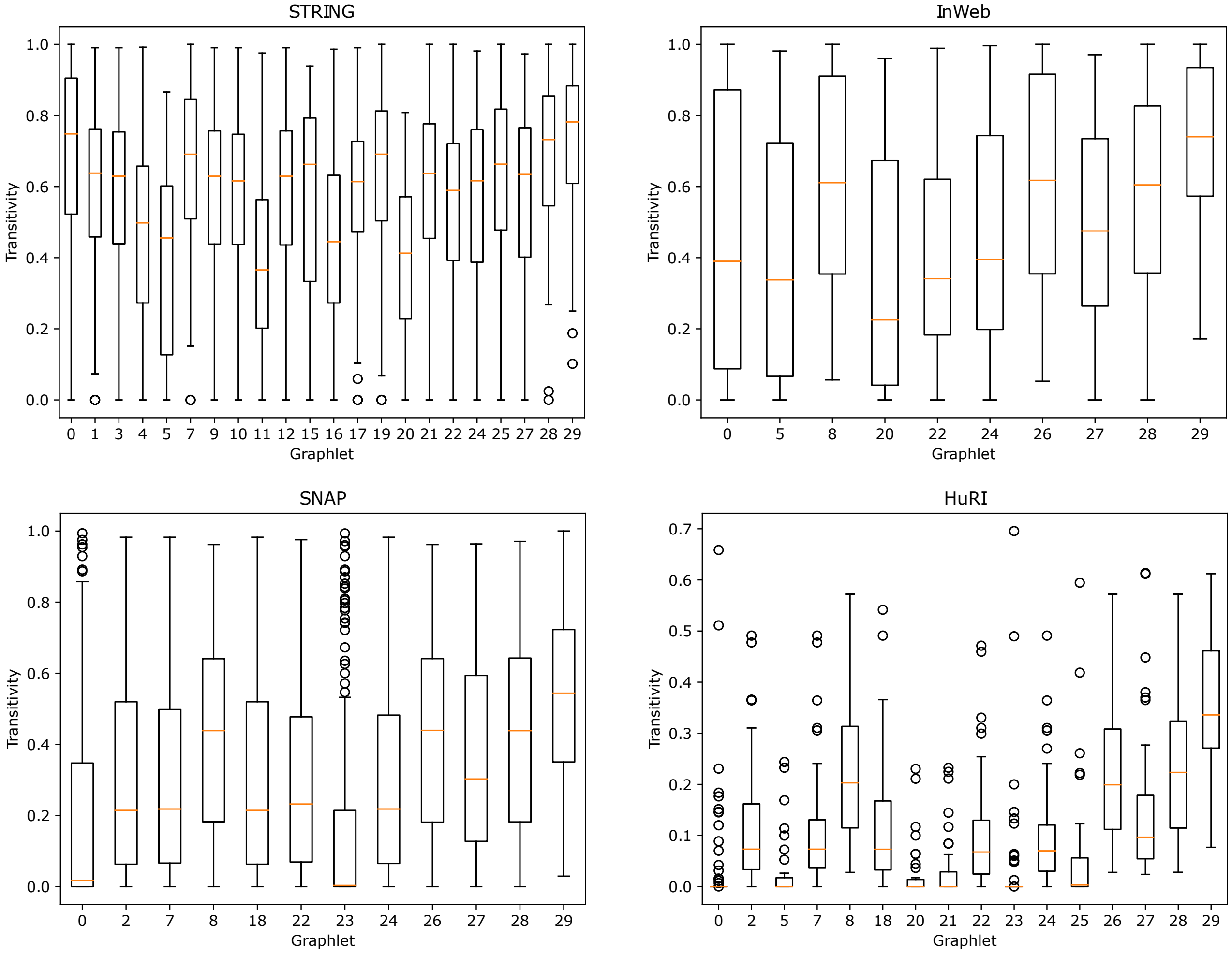

Figure S6: Boxplots showing the distribution of transitivity (defined as the ratio of number closed triads in a network to the total number of triads) in clusters obtained by different non-redundant graphlets in each interactome. For each cluster, we first find the graph induced by the nodes in that cluster and then compute the transitivity of the induced graph.

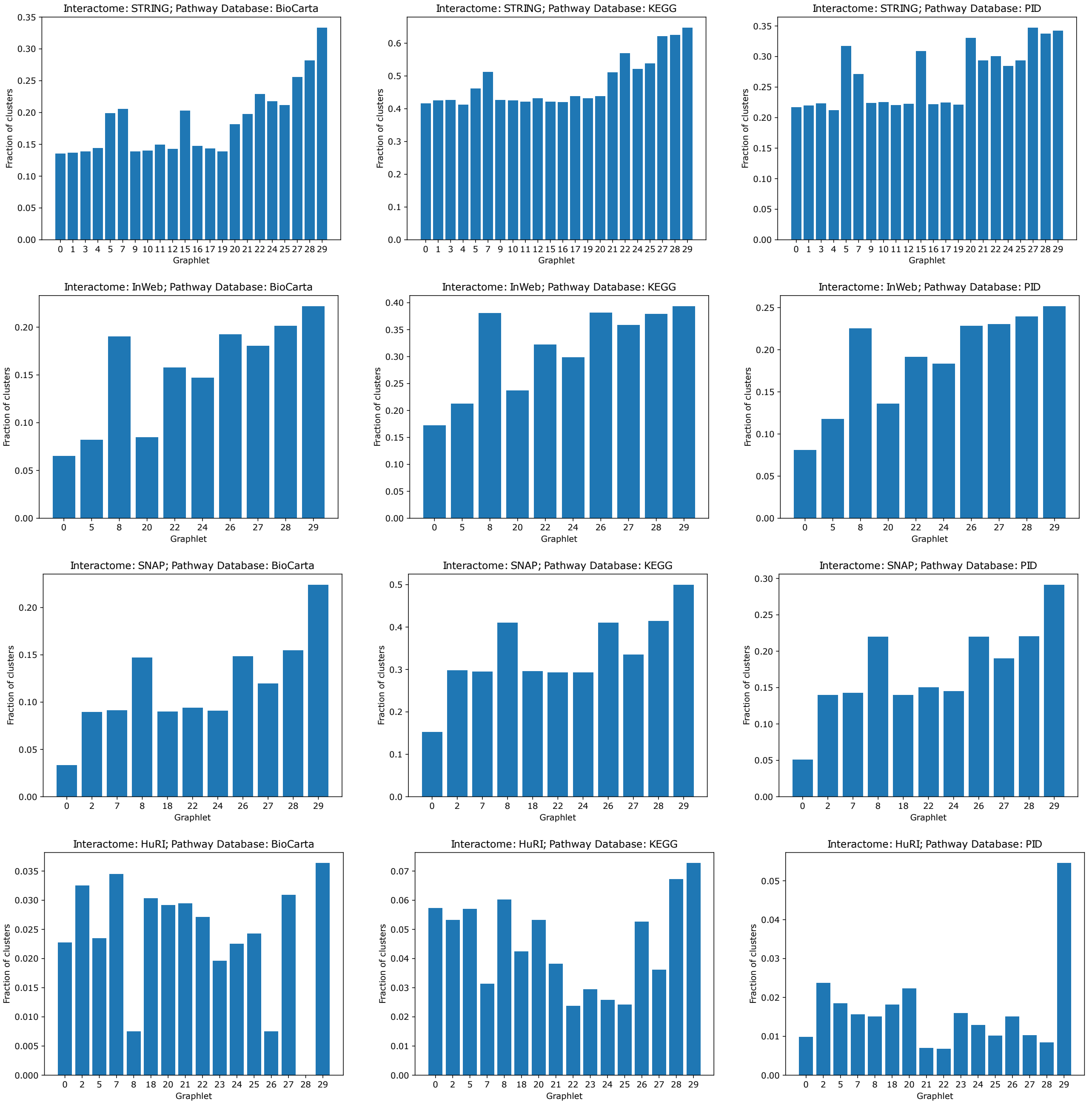

Figure S7: Fraction of significantly enriched clusters for each (non-redundant) graphlet-based clustering in all four interactomes considered.

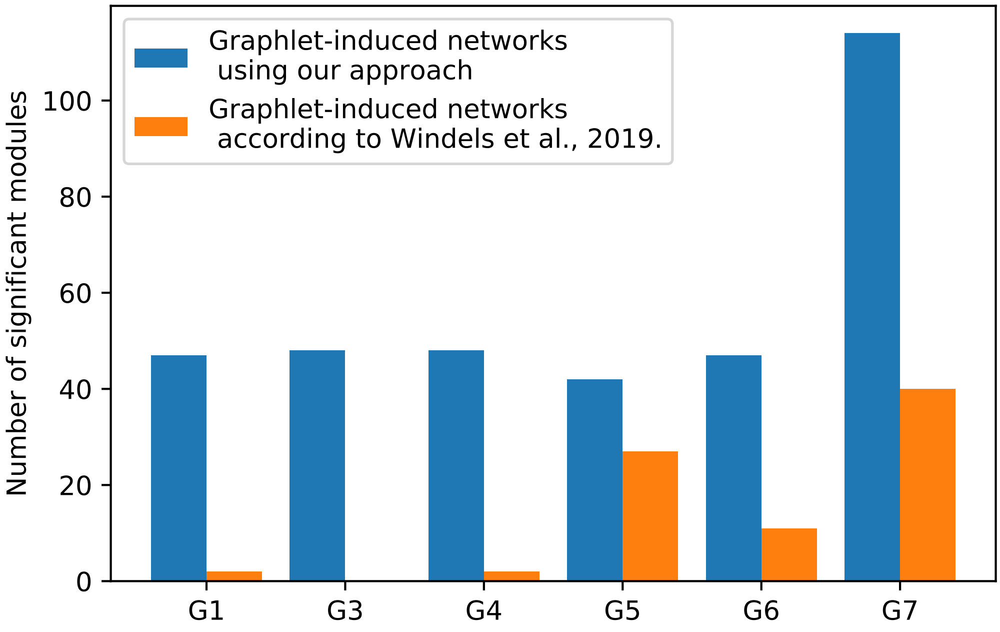

Figure S8: Number of significant modules in two different graphlet-based module detection methods in the SNAP interactome with SNAP disease association dataset for upto four node graphlets. Our approach retains the links that participate in the given graphlet while discarding the rest whereas Windels et al. considers adding links between any pair of nodes that are part of the same graphlet. It shows that our approach finds more significant modules. We only show induced networks that are different in the two methods as for G0, G2, and G8, the two methods are equivalent.

Disease	Best adj. p-value
Thrombosis	0.09
Chronic Myeloid Leukemia	1.0
Age related macular degeneration	0.08
Glioblastoma	1.0

Table S2: Table of p-values of G_0-based clustering in SNAP and selected DisGeNET associations as shown in Fig. 6 (main text).

Trait	Category	GWAS name	Reference
Coronary Art. Dis.	Cardiovascular	EUR.ASN.CAD.cad.add.160614.website.txt.tgz	Nikpay et al., *Nat Gen* 2015.
Body Mass Index	Anthropometric	EUR.BMI.ENGAGE1000G_BMI.txt.tgz	Horikoshi et al., *PLoS Gen* 2015.
Type 2 Diabetes	Glycemic	EUR.DIAGRAMv3.2012DEC17.T2D.txt.gz	Morris et al., *Nat Gen* 2012.
Overweight	Anthropometric	EUR.GIANT_OVERWEIGHT_Stage1_ Berndt2013_publicrelease_HapMapCeuFreq.txt.gz	Berndt et al., *Nat Gen* 2013.
Alzheimer's Disease	Neurodegenerative	EUR.IGAP_stage_1.txt.gz	Lambert et al., *Nat Gen* 2013.

Table S3: Table of GWAS traits and names.

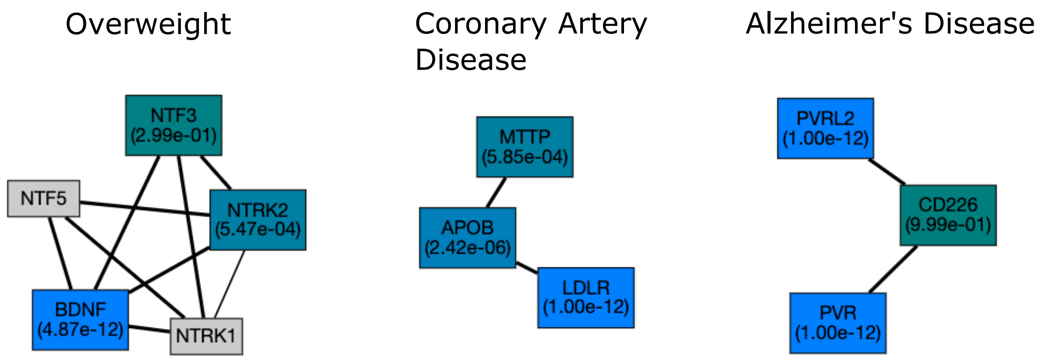

Figure S9: Additional GWAS-trait associated significant modules found by higher-order graphlets that are not found either by G_0-based clustering or by one of the top 5 methods from the DREAM challenge. The associated traits Overweight, Coronary Artery Disease, and Alzheimer's Disease are detected by graphlets G_{24}, G_{22}, and G_5 respectively. The lighter shades of blue represent smaller gene p-values.
